# Magnetism and local symmetry breaking in a Mott insulator with strong spin orbit interactions

**DOI:** 10.1038/ncomms14407

**Published:** 2017-02-09

**Authors:** L. Lu, M. Song, W. Liu, A. P. Reyes, P. Kuhns, H. O. Lee, I. R. Fisher, V. F. Mitrović

**Affiliations:** 1 Department of Physics, Brown University, Providence, Rhode Island 02912, USA; 2 National High Magnetic Field Laboratory, Tallahassee, Florida 32310, USA; 3 Department of Applied Physics and Geballe Laboratory for Advanced Materials, Stanford University, Stanford, California 94305, USA; 4 Stanford Institute for Materials and Energy Sciences, SLAC National Accelerator Laboratory, 2575 Sand Hill Road, Menlo Park, California 94025, USA

## Abstract

Study of the combined effects of strong electronic correlations with spin-orbit coupling (SOC) represents a central issue in quantum materials research. Predicting emergent properties represents a huge theoretical problem since the presence of SOC implies that the spin is not a good quantum number. Existing theories propose the emergence of a multitude of exotic quantum phases, distinguishable by either local point symmetry breaking or local spin expectation values, even in materials with simple cubic crystal structure such as Ba_2_NaOsO_6_. Experimental tests of these theories by local probes are highly sought for. Our local measurements designed to concurrently probe spin and orbital/lattice degrees of freedom of Ba_2_NaOsO_6_ provide such tests. Here we show that a canted ferromagnetic phase which is preceded by local point symmetry breaking is stabilized at low temperatures, as predicted by quantum theories involving multipolar spin interactions.

## Abstract (web summary)

Local probing into the microscopic degrees of freedom is highly desired to understand emergent exotic quantum phases. Here, the authors report a canted ferromagnetic phase preceded by local point symmetry breaking at low temperature in Ba_2_NaOsO_6_, acquired by probing into the spin, orbital and lattice degrees of freedom.

Magnetic Mott insulators with strong spin-orbit coupling (SOC) represent an intriguing class of materials where various exotic quantum phases, that include spin liquid, multipolar charge order, topological insulator and semimetal, Weyl semimetal and Axion insulator, are predicted to emerge^1^,^2^,^3^,^4^,^5^,^6^,^7^,^8^,^9^,^10^. SOC is a relativistic effect that in the strong regime leads to local entanglement of spin and orbital degrees of freedom. This entanglement results in drastically different physics than in cases of weak SOC. For example, strong SOC can significantly enhance quantum fluctuations. The associated emergent phenomena are particularly rich in metal oxides containing 5*d* transition-metal ions owing to the comparable magnitude of both strong electron correlations and SOC. Particularly interesting is the case of materials with a double perovskites structure^11^,^12^,^13^,^14^,^15^, for which it has been proposed that partial lifting of degeneracy of the total angular momentum eigenstates induces a highly nontrivial multipolar exchange interactions^3^,^4^. These peculiar interactions promote quantum fluctuations and thus generate novel quantum states impossible without strong SOC^3^,^4^,^16^. Such states include an unconventional antiferromagnet with the dominant magnetic octupole and quadrupole moments, an unusual noncollinear ferromagnet with a doubled unit cell and magnetization along the [110] axis, and biaxial spin nematic phase with quadrupolar order with preserved time-reversal symmetry stabilized in a broad intermediate temperature range above any magnetic ordering temperature. A key feature of these many-body quantum models is that significant interactions are fourth and sixth order in the effective spins, due to strongly orbital-dependent exchange.

A representative material in this class is Ba_2_NaOsO_6_, a double perovskite with Na and Os ions inhabiting alternate cation B sites, which for an undistorted structure has a face-centered-cubic lattice, as shown in Fig. 1a. Thermodynamic and reflectivity measurements characterize this material as a 5*d*^1^ ferromagnetic (FM) Mott insulator with a moderate ordered moment per formula unit and *T*_c_∼6.3 K (refs 12, 17). This relatively small value of the ordered moment was confirmed in μSR measurements^18^. Taking SOC into account, the anticipated ground state for a perfectly cubic point symmetry is *J*_eff_=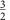. Yet, the magnetic entropy removed at FM transition is only *R* ln 2 (ref. 12). Alhough, the most unusual observation is that the FM state easy axis is in the [110] direction, as this does not occur in standard Landau theory for ferromagnetism in a cubic symmetry^3^. This uncommon magnetism can either be explained by the density functional theory electronic structure calculations, that include effects of electron correlation, a strong SOC, and anisotropic exchange interaction^19^; or, by quantum models including multipolar exchange interactions arising from strong SOC^3^,^20^. Moreover, quantum models identify the quadrupolar/orbital ordering, as a driving mechanism for the FM phase that develops atop it^3^. This quadrupolar order is characterized by the orbital polarization that is distinct on the two sublattices. As such polarizations cannot be time-reversal conjugates, when magnetism onsets, a net FM moment results. Moreover, because this anticipated quadrupolar ordering, that manifests in a breaking of the local cubic symmetry, onsets at higher temperature than magnetic order, many-body quantum based models also account for the missing entropy at the FM transition^3^,^12^ (see Supplementary Discussion 3). Experimental confirmation of the microscopic quantum models requires the observation of two effects. These comprise a structural change, that precedes magnetic order, associated with the quadrupolar ordering and local spin expectation values, that differ from the average ones. Here we report the first observation of such effects. Namely, we observe exotic local spin expectation values along with the structural changes, and infer the exact microscopic nature of the FM state and lattice distortions. We show that the FM state is in fact a type of canted ferromagnet with two sub-lattice magnetization, and that cubic symmetry breaking occurs at a temperature above the Néel temperature and it involves deformation of oxygen octahedra presumably reflecting a complicated pattern of staggered orbital order. In light of our NMR findings, we compiled the phase diagram sketched in Fig. 1b. Our results are in startlingly good agreement with recent theoretical predictions based on quantum models^3^,^20^. Thus, our findings establish that such quantum models represent an appropriate theoretical framework for predicting emergent properties in materials with both strong correlations and SOC, in general.

## Results

### NMR spectra

**Figure 1:**
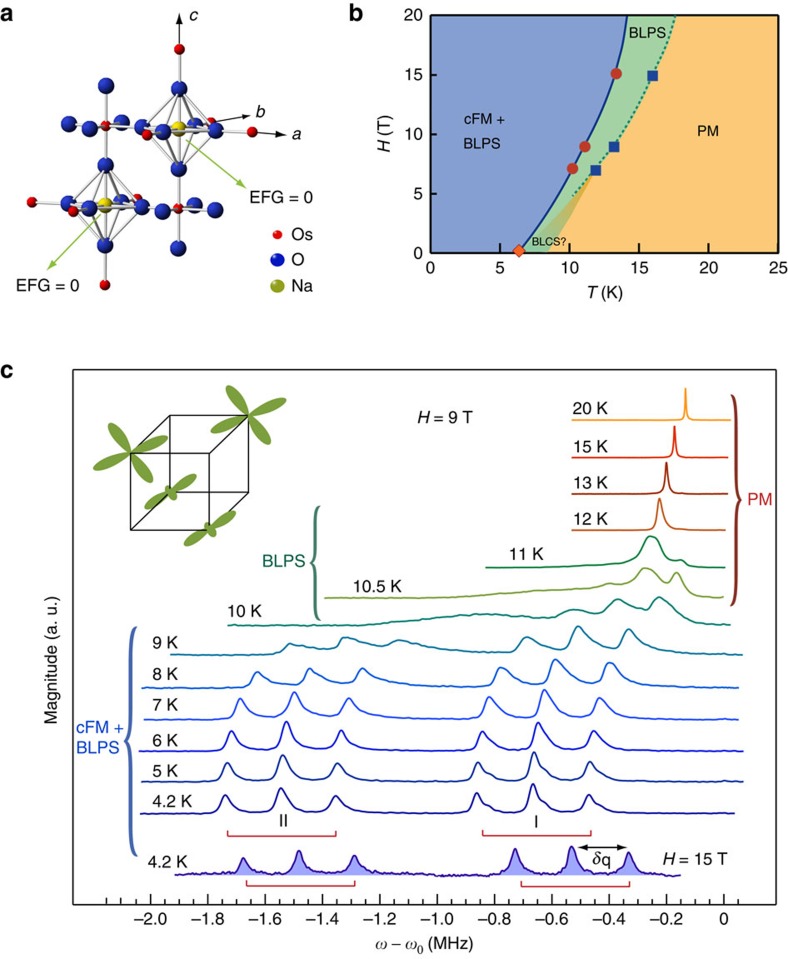
Phase diagram of Ba_2_NaOsO_6_ deduced from the NMR spectra. (**a**) The high-temperature undistorted crystal structure of Ba_2_NaOsO_6_. In this case, point symmetry at the Na site is cubic leading to zero electric field gradient (EFG). Principal crystallographic axes are shown as well. (**b**) Sketch of the phase diagram based on our NMR measurements. Squares indicate onset temperature for the local cubic symmetry breaking, determined from our NMR data as explained in the text, in the paramagnetic (PM) phase. Circles denote *T*_c_, transition temperature into canted ferromagnetic (cFM) phase, as deduced from the NMR data, while diamond marks *T*_c_ as determined from thermodynamic measurements in ref. 12. The solid line indicates phase transition into cFM state and also possible tetragonal-to-orthorhombic phase transition. The dashed line denotes cross over to the broken local point symmetry (BLPS) phase, as detected by NMR. (**c**) Temperature evolution of ^23^Na spectra at 9 T (and at 15 T, shaded trace) magnetic field applied parallel to [001] crystalline axis. Narrow single peak spectra characterize high-temperature paramagnetic (PM) state. At intermediate temperatures, broader and more complex spectra reveal the appearance of electric field gradient (EFG) induced by breaking of local cubic symmetry. Splitting into two sets of triplet lines (labelled as I and II), reflecting the existence of two distinct magnetic sites in the lattice, is evident at lower temperatures. Zero of frequency is defined as *ω*_0_=^23^*γH*. PM, paramagnetic; BLPS, broken local cubic symmetry; and cFM, canted ferromagnetic. The charge density in the theoretically predicted quadrupolar phase^9^ is sketched in the inset.

In a lattice with cubic symmetry only one Na NMR site is present, which results in a narrow single peak spectrum in the insulating paramagnetic (PM) state. Our main discovery is that, on reducing temperature, the Na NMR line undergoes a complex modification, as evident in Fig. 1c, reflecting changes in local electron spin susceptibility and electric field gradient (EFG). That is, we deduce that this modification reflects a splitting of Na into two sites sensing different hyperfine fields due to electronic spins and breaking of the local cubic point symmetry with both sites sensing the same quadrupole frequency, related to the EFG.

To establish the sensitivity of our measurements to putative lattice distortions, orbital order, and magnetism, we first inspect the temperature (*T*) dependence of ^23^Na NMR spectra in Ba_2_NaOsO_6_. These ^23^Na spectra reveal the distribution of the hyperfine fields and the electronic charge and are thus a sensitive probe of both the electronic spin polarization (local magnetism) and charge distribution (orbital order and lattice symmetry). Temperature evolution of ^23^Na NMR spectra is plotted in Fig. 1c. For *T*>12 K, the spectrum consists of a single narrow NMR line, evidencing the PM state. Below 13 K, the NMR line broadens and splits into multiple peaks indicating onset of significant changes in the local symmetry, thereby producing EFG, that is, asymmetric (non-cubic) charge distribution. Below 10 K, the ^23^Na spectra clearly split into six peaks, that is, two sets of triplet lines, labelled as I and II in Fig. 1c, that are well separated in frequency.

### Two magnetic sites

Broadly speaking, the emergence of these two sets of triplets indicates appearance of two distinct magnetic sites, that is, two nuclear sites that sense two different local fields, in the lattice. In a field of 9 T, the transition to the long range ordered (LRO) state onsets in the vicinity of 10 K, which is significantly higher than the transition temperature observed in low-field thermodynamic measurements in ref. 12. Our data measured at the different applied magnetic fields indicate that the transition temperature increases with the increasing field, confirming the magnetic nature of the transition (see Supplementary Note 2). The line shape and the fact that the two sets of triplet lines, I and II, are well separated in frequency implies that the low-temperature LRO magnetic state is commensurate. Furthermore, both sets of lines are shifted to frequencies below that of spectra in the PM state. This demonstrates that the net local magnetic fields on both Na sites are of the same sign indicating that the LRO order is likely ferromagnetic.

### Point symmetry breaking and EFG

Besides the splitting into sets I and II, that reflects the appearance of two distinct magnetic sites in the low *T* phase, we observe additional splitting of each set of the spectral lines into three peaks. Moreover, as visible in Fig. 1c, the additional splitting is discernible at temperatures higher than that for the onset of LRO state, apparent in emergence of sets I and II. This splitting, labelled as *δ*_q_ in Fig. 1c, originates from quadrupole interaction, implying changes in local charge distribution induced by modifications of electronic orbitals and/or local lattice symmetry.

**Figure 2:**
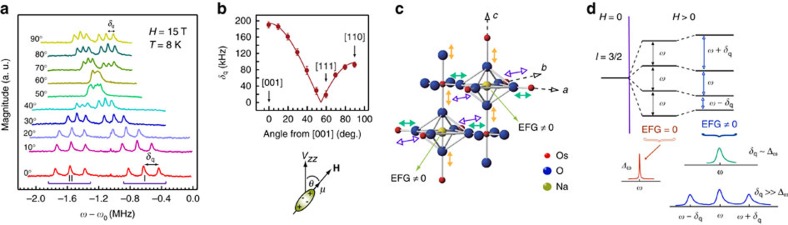
Local cubic symmetry breaking in the ordered phase. (**a**) ^23^Na spectra in low temperature ordered state as a function of the angle between the applied magnetic field and [001] crystalline axis. In this case, **H** was rotated in the (1
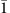
0) plane of the crystal which contains three high symmetry directions: [001], [111] and [110]. (**b**) The mean peak-to-peak splitting (*δ*_q_) between any two adjacent peaks of the triplets I and II. Error bars reflect the scattering of deduced (*δ*_q_) values. Solid line is the fit to 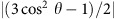, where *θ* denotes the angle between the principal axis of the EFG (*V*_*zz*_) and the applied magnetic field (**H**) as depicted for nuclei with spin *I*=3/2 and magnetic moment **μ** (see Supplementary Note 5). (**c**) Schematic of a proposed lattice distortion involving the O^2−^ ions. The distortion breaks cubic symmetry at the Na site giving rise to finite electric field gradient (EFG). Different types and colours of double arrows illustrate unequal magnitude of oxygen displacements. Displacements along different axes can comprise either from compression or elongation of the oxygen bonds (see Supplementary Note 5). Depicted distortion magnitude is not to scale with the distance between nearest neighbour Os atoms, and is amplified for clarity. (**d**) Schematic of the energy levels of a spin-3/2 nucleus in a finite magnetic field and in the presence of quadrupolar interaction with EFG, generated by surrounding electronic charges, and resulting NMR spectra. In the absence of quadrupolar interaction spectrum consists of a single narrow line at frequency *ω* and of width Δ_*ω*_. In the presence of quadrupolar interaction, the centre transition remains at frequency *ω*, while the satellite transitions appear at frequencies shift by ±*δ*_q_ , proportional to the magnitude of the EFG. For small values of the EFG, satellite transition cannot be resolved and only line broadening is observed. Strictly speaking, there is also a broadening due to the distribution of magnitude of the EFG itself, but this is manifested only on the satellites and not on the central transition. In our case, this distribution can be neglected as all the lines show the same width.

For nuclear sites with spin *I*>1/2, such as ^23^Na with *I*=3/2, and non-zero EFG, quadrupole interaction between nuclear spin and EFG splits otherwise single NMR line to 2*I* lines, as illustrated in Fig. 2d. Thus, ^23^Na spectral line splits in three in the presence of non-zero EFG. Nonetheless, for small finite values of the EFG three peaks are not necessarily discernible, in which case significant line broadening can only be observed, as depicted in Fig. 2d. At sites with cubic point symmetry the EFG is zero (see Supplementary Note 5), as is the case for Na nuclei in the high temperature PM phase. Therefore, the observed line broadening and subsequent splitting of the Na spectra into triplets, in the magnetically ordered phase, indicates breaking of the cubic point symmetry, caused by local distortions of electronic charge distribution. These distortions, marking the broken local point symmetry (BLPS) phase, occur above the transition into the magnetic state, as depicted in Fig. 1b. To confirm this finding, we measured low *T* spectra as a function of strength and orientation of the applied magnetic field, as we describe next.

Specifically, we examine *δ*_q_, the average separation between two adjacent quadrupolar satellite lines, as a function of strength (*H*) and orientation (**H**) of the applied magnetic field. The size of *δ*_q_ is proportional to the magnitude of the EFG and the square of the spin operator 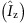 projected along the principal axis of the EFG (*V*_*zz*_) (see Supplementary Note 5). In strong applied field, as is the case in our experiment, the size of *δ*_q_ is controlled by the projection of 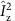 along **H**, proportional to the cos^2^ (*θ*) of the angle *θ* between **H** and *V*_*zz*_, as shown in Fig. 2b. Evidently, *δ*_q_ is at its maximum for **H** applied in the direction of *V*_*zz*_, which in our experiment corresponds to [001] direction. Therefore, if the splitting *δ*_q_ originates from quadrupole interactions, *δ*_q_ should remain constant as strength of the the applied field is changed and should follow 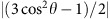 function of *θ* as its orientation is varied^21^. We compare *δ*_q_ in fields ranging from 7 to 15 T at 4 K, deep in the LRO phase (see Fig. 1c). Comparison reveals that *δ*_q_ varies by <2%, which is of the order of the error bars. Furthermore, we measured the spectra at 15 T and 8 K as a function of the angle (*θ*) between **H** and [001] crystalline axis as plotted in Fig. 2a. The angle dependence of the splitting *δ*_q_ is displayed in Fig. 2b. It indeed follows the exact functional dependence expected for the *δ*_q_ originating from quadrupole interactions. Both the observed insensitivity of *δ*_q_ to the strength of the magnetic field and its dependence on *θ* indicate that *δ*_q_ splitting originates from structural/orbital distortions for which the principal axes of the EFG coincide with those of the crystal (see Methods). Moreover, the insensitivity of *δ*_q_ to the strength of the magnetic field rules out the possibility that the detected distortions originate from trivial magnetostriction effects on the crystal (see Supplementary Discussion 2). Our finding, that structural distortion is present in the LRO phase, is in contrast to the predictions made by the first-principles density functional theory calculation^19^,^22^. This is important in so far that it clearly shows that quantum models based on complex multipolar interaction generating high-order spin exchange is consistent with the observed nature of emergent phases in Mott insulators with the strong SOC^3^,^20^.

### Lattice distortions and orbital order

To resolve the microscopic nature of the observed lattice distortions and determine their magnitude, we performed detailed numerical calculations of the EFG, and thus *δ*_q_, based on the point charge approximation^21^ (see Supplementary Note 5). We found that our observation, revealing equal *δ*_q_ on two magnetically inequivalent Na sites, can be best explained by a scenario involving distortions of the O^2−^ octahedra, surrounding Na^+^ ions as depicted in Fig. 2c. In this scenario, one structurally distinct Na site in non-cubic environment is generated. As it results from our calculations, distortions that generate orthorhombic local symmetry at the Na site are required to account for both the amplitude of the detected splitting (*δ*_q_≈190 kHz) and its dependence on the field orientation. Thus, in the ordered phase, our observations are explained by the orthorhombic distortions that comprise of dominant deformations along [001] and one of the crystalline axis in the (110) plane. Even though we find several possible distortions which can induce the observed splitting, we emphasize that they all involve a symmetry-lowering transition to an orthorhombic point symmetry in the LRO phase. Considering the observed amplitude of *δ*_q_, we deduce that a typical magnitude of the distortion along any particular direction in the LRO phase does not exceed 0.8% of the respective lattice constant. Above *T*_c_, in the BLPS phase, the width of the NMR spectra allows us to place an upper limit on distortions. We infer that the limit equals to 0.02% of the respective lattice constant, as any deformations that exceed this value would cause visible splitting of the NMR spectra in the PM state.

In the PM state, BLPS phase is characterized by significant broadening of the NMR spectra. This broadening grows rapidly on decreasing temperature towards *T*_c_. The angle dependence of the broadening does not coincide with either that of the internal uniform or staggered fields, indicating that the broadening predominantly originates from lattice distortions. Because in the BLPS phase we do not observe well defined splitting, but rather convoluted broadening, the exact dependence of *δ*_q_ on the field orientation is unknown. Consequently, dominant tetragonal distortions along [001] direction can in principle account for the line broadening in the PM phase (see Methods). Thus, the BLPS phase can be viewed as the PM phase in which the cubic point symmetry is broken by either dominant tetragonal deformations of the oxygen tetrahedra along [001] direction or orthorhombic distortions, as is the case in the LRO phase (see Supplementary Note 5 and Supplementary Discussion 1). Hence, it is possible that the solid line, indicating *T*_c_ into LRO magnetic phase, in Fig. 1b denotes symmetry lowering transition from tetragonal-to-orthorhombic phase as well.

### LRO magnetic state

**Figure 3:**
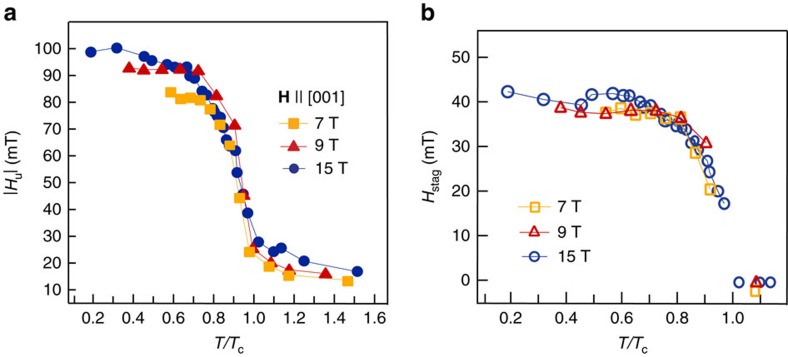
Temperature dependence of the uniform and staggered fields. Absolute value of the uniform internal field, *H*_u_, (**a**) and the staggered internal field, *H*_stag_, (**b**) as a function of reduced temperature for various 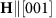. Typical error bars are on the order of a few per cent and not shown for clarity. Lines are guide to the eyes.

**Figure 4:**
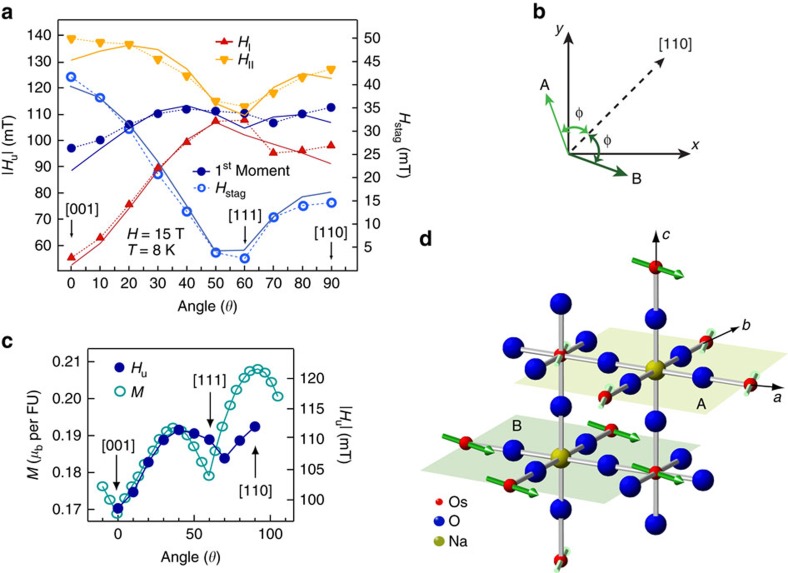
Resolution of the spin orientation in the ordered phase. (**a**) The magnitude of the internal field associated with triplet I (*H*_I_) and II (*H*_II_) (solid symbols), and the first moment of the entire spectrum (uniform field, *H*_u_) as a function of the angle between the applied magnetic field and [001] crystalline axis at 8 K and 15 T. Open symbols depict the angular dependence of the staggered field, *H*_stag_. Typical error bars are on the order of a few per cent and not shown for clarity. Dotted lines are guide to the eyes. Solid lines are calculated fields from the spin model sketched in part (**d**), as described in the text. (**b**) Schematic of the net magnetization in the XY plane in the spin model, consistent with our data and proposed in ref. 20. Arrows of different shades depict spins from two sub-lattices, labelled as A and B. These spins of equal magnitude are canted by angle ±*φ* with respect to [110] direction. (**c**) Comparison of the angular dependence of bulk magnetization per FU (formula unit) from ref. 12 (open symbols) and *H*_u_ determined from our NMR measurements. Lines are guide to the eyes. (**d**) Schematic of the spin model consistent with our data and proposed in ref. 20. Different shades and orientation of arrows indicate distinct ionic (spin) environments on Os site. Planes containing moments from sub-lattice A and B are shown. These equal magnitude moments in two sub-lattices are canted with respect to [110] direction.

We emphasize that detected lattice distortions lead to only one magnetically distinct Na site. Thus, we conclude that the observed magnetically distinct Na sites (labelled as I and II in Fig. 1) must originate from the novel type of magnetism and not from trivial artifacts of lattice distortions. In order to deduce the microscopic nature of the LRO magnetism, we next discuss the temperature and field evolution of the local fields. We used the NMR shift data to infer the local uniform 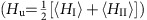 and staggered 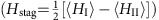 fields, where the average is taken over the triplet I and II, as denoted (see Supplementary Notes 1 and 2). We note that *H*_u_ corresponds to the local field as determined by the first moment of the entire spectra. Temperature evolution of these local fields is displayed in Fig. 3. Well below *T*_c_, *H*_u_ increases with increasing *H*, while *H*_stag_ (observable only by local probes) remains constant. Interestingly, both *H*_u_ and *H*_stag_ are of the same order of magnitude. Presence of *H*_stag_ implies that the LRO state contains two-sublattice magnetization with significant antiparallel components. Such magnetization naturally accounts for the appearance of two magnetically inequivalent Na sites, that is the appearance of distinct local fields 〈*H*_I_〉 and 〈*H*_II_〉. As visible in Fig. 4a, the internal field at the Na site consists of a sum of projection along **H** of total of six nearest-neighbour Os moments, four of which have equal magnetic moment projections as they belong to the same sub-layer. That is, the internal field at the Na site in one plane consists of a sum of the projection of the four Os moments on the same layer, and thus equal projected moments from one sub-lattice labelled A, and two Osmia above/below in neighbouring layers with projected magnetic moments pointing in a different direction (sub-lattice labelled B), and thus producing different local field than A moments at the Na site. Na nuclei in the next plane will then sense four type B and two type A projected Os moments. This generates two sets of inequivalent Na sites and causes the magnetic splitting in spectrum between triplet I and II, as two types of moments induce different local fields at the Na site.

Next, we inspect the local fields as the applied magnetic field was rotated in the (1
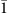
0) plane of the crystal, as illustrated in Fig. 4a. This is essential for ensuring that we understand what components of anisotropic magnetic susceptibility are being measured. For 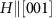, *H*_stag_, as well as 〈*H*_II_〉, reaches its maximum value, while both *H*_u_ and 〈*H*_I_〉 are at their minimum. In principle, *H*_u_ should scale as bulk magnetization (*M*). As evident in Fig. 4c, this is not the case here. This finding reveals that despite the fact that the net magnetization is aligned with the [110] axis, the local fields, that is, spin expectation values, are not. This very fact was predicted to arise as a direct consequence of lattice distortions driven by complex interactions in this class of materials^3^.

The angular dependence of the internal fields is used to deduce the exact spin orientation in the LRO phase by calculating the local *H*_u_ and *H*_stag_ at the Na site for a given spin orientation. The local field consists of a contributions from electronic spins at six nearest-neighbour Os ions mediated via anisotropic hyperfine interaction (Methods). By performing full lattice sum, we calculate the local fields at the Na site as the direction of the applied field is rotated in (1
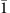
0) plane of the crystal. We find that the model that best describes our observations, as illustrated by the solid lines in Fig. 4a, is a two-sublattice canted FM model, recently proposed in ref. 20 and depicted in Fig. 4b. This model consists of two inequivalent sub-lattices with moments in each layer in the XY plane parallel to each other, forming FM order, while moments in the neighbouring layers point to a different direction. Specifically, moments in two adjacent layers are symmetric about [110] axis, that is they form an angle ±*φ*, with [110] axis, as depicted in Fig. 4b,d. As direction of the applied field is varied spin-plane follows the direction of **H** while spins remain staggered about [110] axis. Moments arranged in this fashion induce an uniform field in [110] direction, providing an overall shift to the NMR spectrum, and form a staggered pattern in the direction perpendicular to [110]. Thus, for **H**‖[001] such spin arrangement generates lowest *H*_u_ and largest *H*_stag_, as observed, when the canting angle *φ* exceeds 45°. The exact value of the canting angle is determined by the fitting procedure described below. The curves in Fig. 4a, are fits to the data using the simulated local fields with relative strength of the off-diagonal terms of the hyperfine coupling tensor, 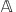, (*A*_*ij*_/*A*_*ii*_), magnitude of the local Os moments, and the canting angle *φ* between the spins on two different sub-lattices, as fitting parameters. Constraining the diagonal terms of 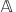 to be close to those found in the PM state, we find the moment of *μ*≈0.6μ_B_ and *φ*≈67°. Using the deduced value of *φ* and formalism in ref. 20, we estimate the ratio of in-plane to intra-plane coupling constant to be ≈4 (see Supplementary Note 4). The value of the moment is in agreement with the effective moment deduced from the fit to a Curie-Weiss law in the PM state in ref. 12. Large canting angle *φ* explains the smaller moment detected in the FM state in bulk measurements^12^,^18^ due to partial cancelation of nonparallel magnetic moments. We emphasize that the symmetry of the inferred 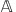 tensor reflects neither local tetragonal nor orthorhombic symmetry of the distorted O octahedra (see Supplementary Notes 3 and 4). This indicates that spin–spin interactions are highly anisotropic.

## Discussion

We have performed microscopic measurements on a model system of Mott insulator with strong SOC, Ba_2_NaOsO_6_. Our static NMR measurements reveal that the local cubic symmetry breaking, induced by deformation of the oxygen octahedra, precedes the formation of the LRO magnetism. Specifically, we find that these deformations generate an orthorhombic point symmetry in the LRO phase. Although, LRO magnetism can also be precedes by tetragonal distortions of the oxygen octahedra. Furthermore, we establish that LRO state is the exotic canted two-sublattice FM state, believed to be driven by the staggered quadrupolar order^3^. This is the first direct detection of such a complex quantum state with the distinct local spin expectation values. Our observation of both the local cubic symmetry breaking and appearance of two-sublattice exotic FM phase is in line with theoretical predictions based on quantum models with multipolar magnetic interactions. The fact that spin–spin interactions are indeed mediated by complex multipolar interactions, as suggested in ref. 3, is further confirmed by our finding that the symmetry of the inferred 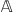 tensor does not reflect any local symmetry of the distorted O octahedra. Moreover, it is proposed that two-sublattice magnetic structure is the very manifestation of staggered quadrupolar order and that this ordering drives the formation of LRO magnetism^3^,^9^. Thus, our finding that LRO phase is a two-sublattice canted FM implies that broken cubic symmetry phase is a staggered quadrupolarly ordered phase with distinct orbital polarization on two-subattices. In summary, our findings clearly demonstrate that microscopic quantum models with multipolar magnetic interactions are an appropriate theoretical framework for predicting emergent quantum phases in Mott insulators with the strong SOC.

Lastly, presented unique direct observation of both local cubic symmetry breaking and exotic LRO magnetic state is the confirmation of the theoretical proposal that the combination of the unusual multipolar interactions, generic for spin-orbitally entangled effective spins, and/or structural transitions or quadrupolar order can lead to a highly frustrated quantum regime even for systems with spin greater than *S*=1/2 (ref. 20). Thus, our work illustrates that such complex quantum states might be found in other frustrated materials with both strong correlations and SOC^23^,^24^.

## Methods

### NMR methods

The measurements were done at Brown University for magnetic field up to 9 T and at the National High Magnetic Field Laboratory (NHMFL) in Tallahassee, FL at higher fields. In both laboratories high homogeneity superconducting magnets were used. The temperature control was provided by ^4^He variable temperature insert. The NMR data were recorded using a state-of-the-art laboratory-made NMR spectrometer. The spectra were obtained, at each given value of the applied field, from the sum of spin-echo Fourier transforms recorded at constant frequency intervals. We used a standard spin echo sequence (*π*/2−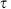
−*π*). Shape of the spectra presented in the manuscript are independent of the duration of time interval 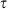. Since nuclear spin *I* of ^23^Na equals to 3/2 and at low temperatures, both Na sites (I and II) are in non-cubic environments, three distinct quadrupolar satellite lines are observed per site^21^. The shift was obtained from the frequency of the first moment of spectral distribution of set of triplet lines using a gyromagnetic ratio of ^23^*γ*=11.2625 MHz/T. The same gyromagnetic ratio was used for all frequency to field scale conversions.

### Sample

High-quality single crystal of Ba_2_NaOsO_6_ with a truncated octahedral morphology were grown from a molten hydroxide flux, as described elsewhere^12^,^17^. Crystal quality was checked by X-ray diffraction, using a Bruker Smart Apex CCD (charge-coupled device) diffractometer, which indicated that the room temperature structure belongs to the *Fm*
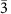
*m* space group^12^. NMR measurements were performed for a single crystal with a volume of ∼1 mm^3^. The quality of the sample was confirmed by the sharpness of ^23^Na NMR spectra both in the high-temperature PM state and low-temperature quadrupolar split spectra.

The sample was both zero-field and field-cooled. We did not detect any influence of the samples cooling history on the NMR spectra. Nevertheless for consistency, all results presented in the paper were obtain in field-cooled conditions. The sample was mounted to one of the crystal faces and rotated with respect to the applied field about an axis using a single axis goniometer. The rotation angle, for applied fields below 9 T, was inferred from the signal of two perpendicularly positioned Hall sensors. In addition, to ensure that data was taken with no external pressure applied, the mounted sample was placed in a solenoid coil with cross sectional area significantly larger than that of the sample. In this way, no pressure is exerted on the sample as coil contracts on cooling.

### Transition temperature

Transition temperature (*T*_c_) from PM to low-temperature FM state was determined by examining the NMR shift and spectral line shapes, as described in detail in Supplementary Note 2. Onset temperature for breaking of local cubic symmetry, shown in Fig. 1b, was identified as temperature below which the second moment of the NMR spectral line, measuring the spectral width, increases notably as compared with that in high-temperature PM phase. We point out that this temperature does not necessarily correspond to the true onset temperature for orbital ordering, which could be undetectable in our experiment due to the subtlety of the effect.

### Quadrupolar interaction and EFG

In the simplest case of a field with axial symmetry, interaction between *eq*, the EFG, and the nucleus, with spin *I* and the quadrupole moment *Q*, is described by the Quadrupole Hamiltonian, 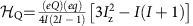. For nuclear spin *I*=3/2, as is the case of ^23^Na, the energy eigenstates of 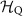 are given by, 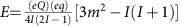. Than, the frequencies between different quadrupole satellite transitions equal,


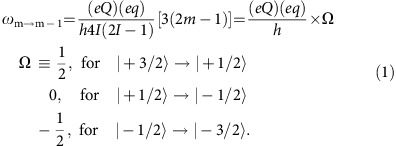


Therefore, in a magnetic field applied along the principal axis of the EFG only three NMR lines (transitions) will be observed with equal splitting *δ*_q_ between adjacent transitions. In this case, the quadrupole splitting *δ*_q_ between different quadrupole satellites is simply given by 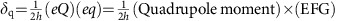. In our experiment, equal splitting is observed between quadrupole satellites lines plotted in Fig. 1c for 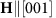 indicating that the principal axis of the EFG must be along **H**, that is along the axis of the crystal. Further, we can estimate the value of the EFG using experimentally determined value of the splitting.

For anisotropic charge distributions, quadrupole Hamiltonian expressed in the coordinate system define by the principal axes of the EFG, is given by


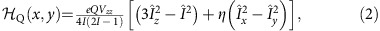


where *η*≡|*V*_*xx*_−*V*_*yy*_|/*V*_*zz*_ is asymmetry parameter and are diagonal components of the EFG. Here, *V*_*zz*_ is defined as the principle component of the EFG and |*V*_*xx*_|<|*V*_*yy*_|<|*V*_*zz*_|, by convention. In this case, the splitting between the adjacent transitions is given by, 
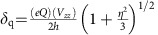
. Thus, the value of *δ*_q_ is dictated by both *V*_*zz*_ and anisotropy parameter. In the high field limit, when 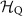 is a perturbation to the dominant Zeeman term, the angular dependence of the splitting is given by


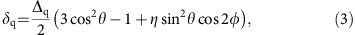


where *θ* is the angle between the applied field **H** and *V*_*zz*_. As in the case of axially symmetric EFG, in the coordinate system defined by the principal axes of the EFG only three NMR lines (transitions) will be observed with equal splitting *δ*_q_ between any adjacent lines. When **H** is rotated in such coordinate system only three NMR lines are observed and the magnitude of the splitting between the adjacent lines depends only on angle *θ*. The fact that we observe no more than three lines per set (I or II) regardless of the angle between **H** and [001] crystalline axis, as shown in Fig. 2b, indicates that **H** was rotated in the coordinate system defined by the principal axes of the EFG. Therefore, the principal axes of the EFG must coincide with those of the crystal.

In a material with cubic symmetry, it is thus possible to stabilize three different domains, each with the principle axis of the EFG, *V*_*zz*_, pointing along any of the three equivalent crystal axes. Further, local magnetic field has to be parallel to *V*_*zz*_ in each domain. The facts that the splitting is the largest for 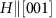 (Fig. 2b), and that only three peaks per set are observed for 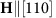 imply that two domains are plausible in the crystal. One domain is characterized by pure uniaxial 3*z*^2^−*r*^2^ distortions where *V*_*zz*_ is in [001] direction, while the other is distinguished by *x*^2^−*y*^2^ distortions where *V*_*zz*_ is then in the (110) plane. In the simplest case *V*_*zz*_ is parallel to [001] direction with *η*=0 indicating tetragonal local symmetry. In the second case, *V*_*zz*_ is aligned along [100] direction with *η* of the order of 1, implying orthorhombic local symmetry. To determine the exact local symmetry, splittings *δ*_q_ obtained for **H** rotated about one of the crystalline axis and about [110] direction have to be analysed.

### Calculation of internal fields

In the LRO phase, the component of the internal hyperfine field parallel to **H**, at an Na site, is given by 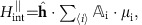 where 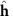 is a unit vector in the applied field direction, 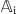 is the symmetric 3 × 3 hyperfine coupling tensor with the nearest-neighbour Os atom and **μ**_i_ is its magnetic moment (see Supplementary Note 4). In the PM phase, hyperfine coupling tensor is diagonal. Due to broken cubic symmetry and complexity of the orbitals mediating the exchange paths, which can induce multipolar exchange interactions^3^,^9^ between neighbouring Os spins, the off-diagonal elements of the hyperfine tensor *A*_*ij*_ are nonzero in the LRO phase. We point out that even if moment is not exclusively localized on Os site but the spin density is distributed to oxygen^19^, our modelling of 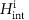 is valid. This is because the complexity of the spin density is accounted for in 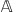. For simplicity, we treat moment as *S*=1/2 localized on Os as was done in ref. 20. By performing full lattice sum, we calculate the local *H*_u_ and *H*_stag_ at the Na site as well as Na NMR spectra, which is a histogram of the local field component projected along the applied field, as the direction of the applied field is rotated in (1
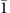
0) plane of the crystal.

## Additional information

**How to cite this article:** Lu, L. *et al*. Magnetism and local symmetry breaking in a Mott insulator with strong spin orbit interactions. *Nat. Commun.* **8,** 14407 doi: 10.1038/ncomms14407 (2017).

**Publisher's note**: Springer Nature remains neutral with regard to jurisdictional claims in published maps and institutional affiliations.

## Supplementary Material

Supplementary Information Supplementary Figures 1-6, Supplementary Tables 1-2, Supplementary Notes 1-5, Supplementary Discussion and Supplementary References

## References

[b1] Chen G., Balents L. & Schnyder A. P. Spin-orbital singlet and quantum critical point on the diamond lattice: FeSc_2_*S*_4_. Phys. Rev. Lett. 102, 096406 (2009). 19392543 10.1103/PhysRevLett.102.096406

[b2] Jackeli G. & Khaliullin G. Mott insulators in the strong spin-orbit coupling limit: from heisenberg to a quantum compass and Kitaev models. Phys. Rev. Lett. 102, 017205 (2009). 19257237 10.1103/PhysRevLett.102.017205

[b3] Chen G., Pereira R. & Balents L. Exotic phases induced by strong spin-orbit coupling in ordered double perovskites. Phys. Rev. B 82, 174440 (2010).

[b4] Chen G. & Balents L. Spin-orbit coupling in *d*^2^ ordered double perovskites. Phys. Rev. B 84, 094420 (2011).

[b5] Pesin D. & Balents L. Mott physics and band topology in materials with strong spin-orbit interaction. Nat. Phys. 6, 376–381 (2010).

[b6] Radić J., Di Ciolo A., Sun K. & Galitski V. Exotic quantum spin models in spin-orbit-coupled mott insulators. Phys. Rev. Lett. 109, 085303 (2012). 23002755 10.1103/PhysRevLett.109.085303

[b7] Cole W. S., Zhang S., Paramekanti A. & Trivedi N. Bose-Hubbard models with synthetic spin-orbit coupling: mott insulators, spin textures, and superfluidity. Phys. Rev. Lett. 109, 085302 (2012). 23002754 10.1103/PhysRevLett.109.085302

[b8] Reuther J., Thomale R. & Trebst S. Finite-temperature phase diagram of the Heisenberg-Kitaev model. Phys. Rev. B 84, 100406 (2011).

[b9] Witczak-Krempa W., Chen G., Kim Y. B. & Balents L. Correlated quantum phenomena in the strong spin-orbit regime. Ann. Rev. Condens. Matter Phys. 5, 57–82 (2014).

[b10] Nussinov Z. & van den Brink J. Compass models: theory and physical motivations. Rev. Mod. Phys. 87, 1–59 (2015).

[b11] Aharen T. . Structure and magnetic properties of the *S*=1 geometrically frustrated double perovskites La_2_LiReO_6_ and Ba_2_YReO_6_. Phys. Rev. B 81, 064436 (2010).

[b12] Erickson A. S. . Ferromagnetism in the Mott insulator Ba_2_NaOsO_6_. Phys. Rev. Lett. 99, 016404 (2007). 17678173 10.1103/PhysRevLett.99.016404

[b13] Yamamura K., Wakeshima M. & Hinatsu Y. Structural phase transition and magnetic properties of double perovskites Ba_2_CaMO_6_ (M=W, Re, Os). J. Solid State Chem. 179, 605–612 (2006).

[b14] WiebeC. R. . Frustration-driven spin freezing in the 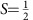 fcc perovskite Sr_2_MgReO_6_. Phys. Rev. B 68, 134410 (2003).

[b15] Wiebe C. R., Greedan J. E., Luke G. M. & Gardner J. S. Spin-glass behavior in the *S*=1/2 fcc ordered perovskite Sr_2_CaReO_6_. Phys. Rev. B 65, 144413 (2002).

[b16] Dodds T., Choy T.-P. & Kim Y. B. Interplay between lattice distortion and spin-orbit coupling in double perovskites. Phys. Rev. B 84, 104439 (2011).

[b17] Stitzer K. E., Smith M. D. & zur Loye H.-C. Crystal growth of Ba_2_MOsO_6_ (M=Li, Na) from reactive hydroxide fluxes. Solid State Sci. 4, 311–316 (2002).

[b18] Steele A. J. . Low-moment magnetism in the double perovskites Ba_2_*M*OsO_6_ (*M*=Li, Na). Phys. Rev. B 84, 144416 (2011).

[b19] GangopadhyayS. & PickettW. E. Spin-orbit coupling, strong correlation, and insulator-metal transitions: The 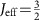 ferromagnetic Dirac-Mott insulator Ba_2_NaOsO_6_. Phys. Rev. B 91, 045133 (2015).

[b20] IshizukaH. & BalentsL. Magnetism in 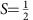 double perovskites with strong spin-orbit interactions. Phys. Rev. B 90, 184422 (2014).

[b21] Abragam A. Principles of Nuclear Magnetism Number 216–263Oxford University Press (1996).

[b22] Xiang H. J. & Whangbo M.-H. Cooperative effect of electron correlation and spin-orbit coupling on the electronic and magnetic properties of Ba_2_NaOsO_6_. Phys. Rev. B 75, 052407 (2007).

[b23] Cook A. M., Matern S., Hickey C., Aczel A. A. & Paramekanti A. Spin-orbit coupled *j*_eff_=1/2 iridium moments on the geometrically frustrated fcc lattice. Phys. Rev. B 92, 020417 (R) (2015).

[b24] Romhànyi J., Balents L. & Jackeli G. Spin-orbit dimers and non-collinear phases in *d*^1^ cubic double perovskites. Preprint at http://arxiv.org/abs/1611.00646 (2016). 10.1103/PhysRevLett.118.217202 28598662

