# Supplementary material for: Magnetism and local symmetry breaking in a Mott insulator with strong spin orbit interactions
Source: Nat Commun. 2017 Feb 9;8:14407. doi: 10.1038/ncomms14407 (PMC5309813; doi:10.1038/ncomms14407)
Supplement: Supplementary Information — Supplementary Figures 1-6, Supplementary Tables 1-2, Supplementary Notes 1-5, Supplementary Discussion and Supplementary References [file ncomms14407-s1.pdf]

SUPPLEMENTARY FIGURES

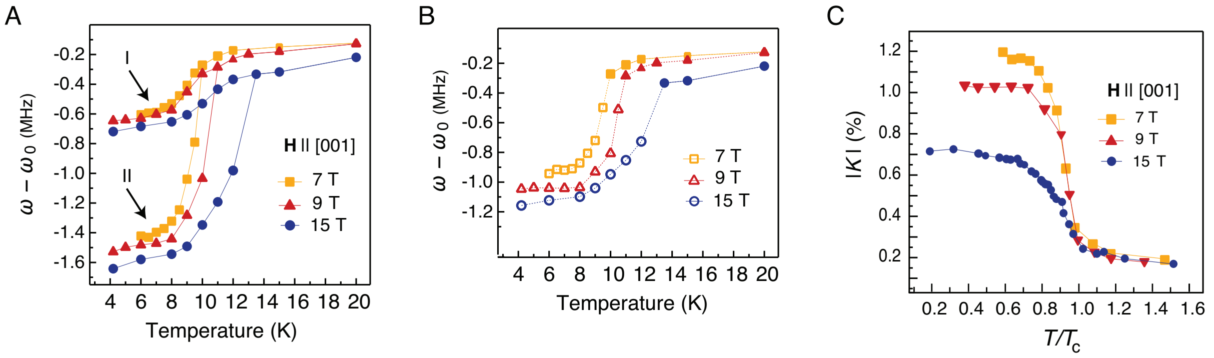

Supplementary Figure 1: Temperature dependence of the shift. **(A)** Temperature dependence of the frequency shift of triplets I and II and the first moment of the entire spectrum **(B)**, at various applied fields. The frequency scale is defined by subtracting ω_0, the zero NMR shift frequency. **(C)** The absolute value of the NMR shift, K, of the first moment the entire spectrum (reflecting uniform spin susceptibility (χ_u^s) as function of reduce temperature for various $\mathbf{H}\|[001]$. The shift is corrected for a small field dependent orbital contribution as determined in the paramagnetic state. Lines are guide to the eyes.

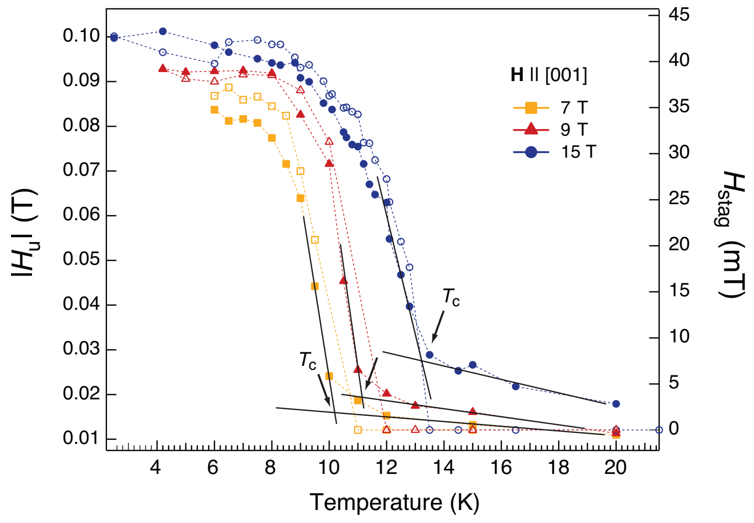

Supplementary Figure 2: Temperature dependence of the uniform and staggered fields. The uniform field data is denoted by filled symbols while open symbols represent staggered fields. Arrows mark transition temperature (T_c) from PM to low temperature magnetic state, determined by the crossing points of the plotted solid lines. These T_c values are displayed in Supplementary Table I. Solid lines are linear fits to the data, while dashed lines are guide to the eyes.

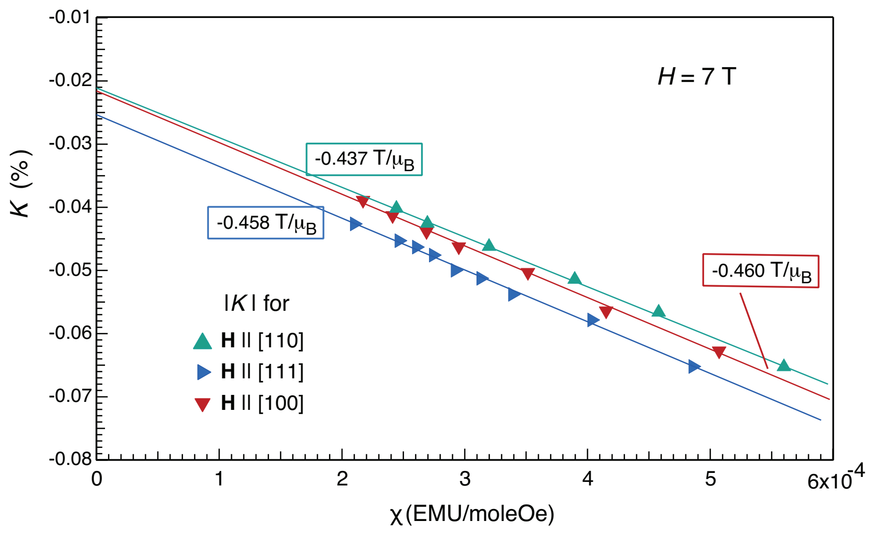

Supplementary Figure 3: ^{23}Na Clogston-Jaccarino plots at 7 T applied field applied along three different directions as denoted. Solid lines are linear fits to the data for temperature ranging from 80 K to 200 K.

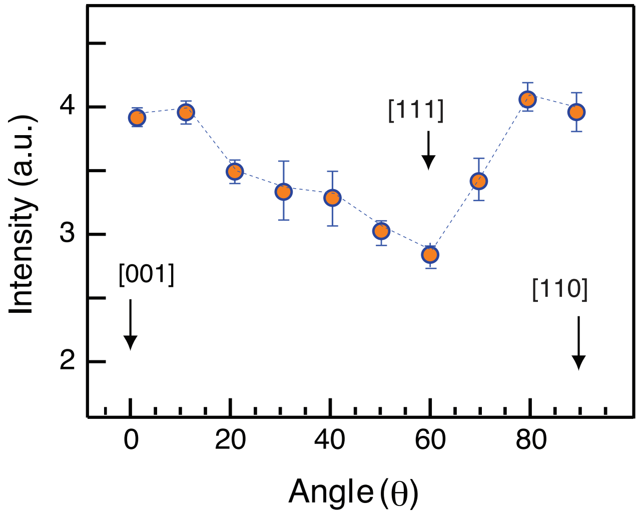

Supplementary Figure 4: Integrated intensity of the entire spectrum, plotted in Fig. 2A of the main manuscript, as a function of the angle between the applied magnetic field and [001] crystalline axis at 8 K and 15 T. Error bars reflect the scattering of deduced intensity values.

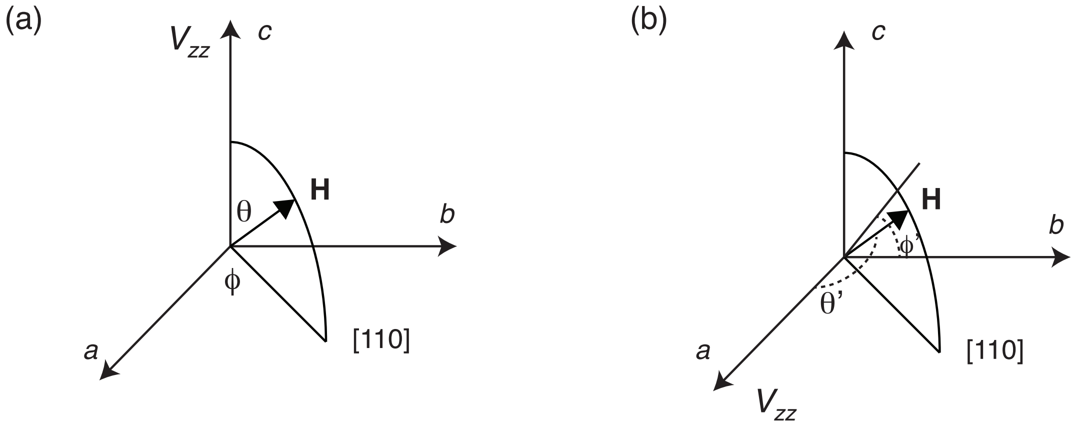

Supplementary Figure 5: Illustration of the rotation of the applied field making angle θ with the respect to the principal axis of the EFG, for: (a) V_{zz} along c-axis of the crystal, (b) V_{zz} along a-axis.

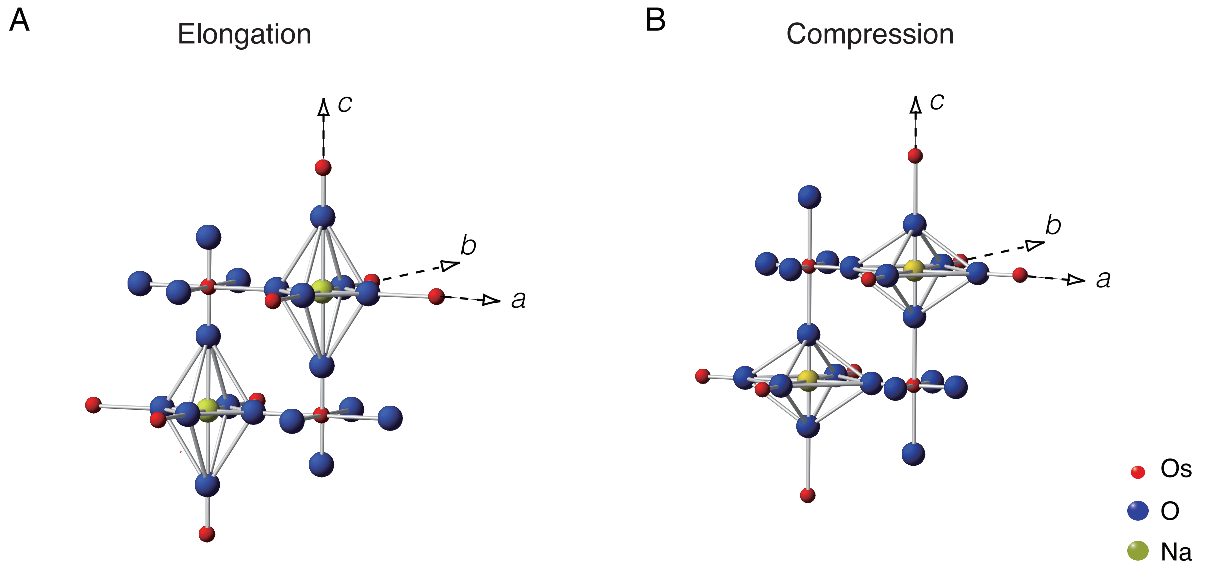

Supplementary Figure 6: Schematic of the proposed lattice distortions that generate one structurally distinct Na site in non-cubic environment by elongation **(A)** or compression **(B)** of one O^{2-} octahedron along [001] direction and its concurrent compression **(A)**, or elongation **(B)**, in [110] plane.

SUPPLEMENTARY TABLES

$\mathbf{H}\parallel[001]$	T_c
0 T	6.3 K [3]
7 T	10.2 K
9 T	11.1 K
15 T	13.3 K

Supplementary Table I: Transition temperature from PM to low temperature magnetic state at various applied fields.

$\mathbf{H}\parallel$	$K_\alpha^{\mathrm{orbit}}$	$g_\alpha A_{\alpha\alpha}(T/\mu_\mathrm{B})$
[001]	-0.0216 %	-0.460
[111]	-0.0253 %	-0.458
[110]	-0.0211 %	-0.437

Supplementary Table II: Hyperfine coupling constants and orbital shifts for different orientations of the applied magnetic field of 7 T for ^{23}Na in Ba_2NaOsO_6.

SUPPLEMENTARY NOTES

Supplementary Note 1: NMR Shift

In Supplementary Figure 1A we plot the temperature dependence of the frequency shift of the first moment of triplets I and II for various strengths of the applied field while that of the first moment of the entire spectra is shown in Supplementary Figure 1B. In addition, we plot temperature variation of the NMR shift ($K \equiv \frac{\omega-\omega_0}{\omega_0}$), quantity proportional to the hyperfine coupling constant (A) and spin susceptibility ($\chi^{\rm s}$), in Supplementary Figure 1C. Because the A for Na is negative, we plot the absolute value of the shift to reflect the spin susceptibility. We observe that below the transition temperature ($T_{\rm c}$) into the ordered state, $\chi^{\rm s}$ decrease with the increasing applied field strength (H).

Supplementary Note 2: Transition Temperature

Transition temperature ($T_{\rm c}$) from paramagnetic (PM) to low temperature ferromagnetic (FM) state was determined by the crossing points of the plotted solid lines in Supplementary Figure 3. The local uniform ($H_u \equiv \frac{1}{2}\left[\langle H_{\rm I}\rangle + \langle H_{\rm II}\rangle\right]$) and staggered ($H_{\rm stag} \equiv \frac{1}{2}\left[\langle H_{\rm I}\rangle - \langle H_{\rm II}\rangle\right]$) fields, where the average is taken over the triplet I and II, were deduced from the shift data shown in Supplementary Figure 1. Evidently, transition temperature increases with increasing applied field. The increase of $T_{\rm c}$ is significant and approximately scales as magnetic energy associated with the applied field. This indeed confirms magnetic nature of the transition.

Supplementary Note 3: Hyperfine coupling tensor for Ba_2NaOsO_6

High Temperature - Paramagnetic Phase:

In our experiment, hyperfine shift at a given temperature is given by,

$$K_\alpha^{\rm hf}(T) = K_\alpha^{\rm orbit} + g_\alpha A_{\alpha\alpha}\chi(T) \tag{1}$$

where $K_\alpha^{\rm orbit}$ denotes temperature independent orbital contribution to the shift, α the principal axis of the shift tensor, $g_{\rm i}$ the electronic g-factor, $A_{\alpha i}$ the transfer hyperfine tensor, and χ electronic susceptibility, *i.e.* $\langle S_{\rm i}\rangle/H$ the net average electronic spin projected along the field direction divided by H. Thus, we determine hyperfine coupling constants and orbital shifts by exploiting the Clogston-Jaccarino type plots [4] of the NMR shift *vs.* bulk susceptibility at a given temperature,

as illustrated in Supplementary Figure 3. More precisely, the slope of the graph is related to the strength of the hyperfine coupling, while the zero intercept gives the orbital shift for a particular orientation of the applied magnetic field. Temperature dependence of the bulk χ can be fitted to the Curie-Weiss behavior with a constant offset, ascribed to Van Vleck paramagnetism, as described in Ref. [3]. In NMR data linear behavior, of the form $K_\alpha^{\rm hf} = K_\alpha^{\rm orb} + m_\alpha \chi(T)$, is found for $T > 25\,$K. Assuming that $K_\alpha^{\rm hf}$ equals to $g_\alpha A_{\alpha\,\alpha}\chi$, we infer the hyperfine coupling constant ($g_\alpha A_{\alpha\,\alpha} = m_\alpha$) in the units of [T/$\mu_{\rm B}$] as shown in Supplementary Table II. Because the lattice structure is cubic in the paramagnetic state, transfer hyperfine tensor, defined in crystalline axes coordinate system, is diagonal with all diagonal elements being equal, *i.e.* $g_\alpha A_{\alpha\alpha} = -0.460\,{\rm T}/\mu_{\rm B}$.

Contribution to the shift from dipole-dipole interaction between Os electronic spin ($S_{\rm N}$) and Na nuclear spin ($\mathbf{I}$) is vanishing due to the cubic symmetry. Breaking of the cubic symmetry that we described in the manuscript is associated with motion of the O ions and does not involve motion of Os ions/spins. Consequently, for the dipole-dipole interaction of this type, cubic symmetry is preserved even in the low temperature magnetic phase. Hence, the contribution of this interaction to the shift is negligible at low temperatures, as well.

Low Temperature - Magnetic Phase:

Assuming that low temperature ordered phase is characterized by distortions of the oxygen octahedra, dominated for example by some small displacement of oxygen ions along $\hat{c}$ axis, a local symmetry at Na site is transformed from cubic, present at high temperatures, to either tetragonal or orthorhombic. The symmetry operation, for example, for tetragonal structure requires the invariance of hyperfine tensor under the fourfold rotation around axis parallel to $\hat{c}$ axis. Requirement that the hyperfine tensor must be invariant under this fourfold rotation, implies the shift tensor $\widetilde{K}$ must posses the following structure

$$\widetilde{K} = \begin{pmatrix} K11 & K12 & 0 \\ -K12 & K11 & 0 \\ 0 & 0 & K33 \end{pmatrix}. \tag{2}$$

This example illustrates that regardless of the exact nature of the low temperature distortions, their dominant effect on $\mathbb{A}$ is to induce finite off-diagonal terms, while changes of diagonal terms are expected to be minor.

Supplementary Note 4: Microscopic Nature of the Magnetic Phase

In order to determine the microscopic nature of the magnetic phase, we calculate the internal hyperfine field generated at the Na site by electronic spins at Os ions. The average internal hyperfine field at the Na site is calculated for different spin configurations and orientation of the applied magnetic field. These results are then compared to the measurements of both internal fields and spectra plotted in Fig. 4A and Fig. 1 of the main paper, respectively. This comparison allowed us to identify possible spin configurations that best describe our observations. We point out that even if moment is not exclusively localized on Os site but the spin density is distributed to O [5], our modeling of $H^{\mathrm{i}}_{\mathrm{int}}$ is valid. This is because the complexity of the spin density is accounted for in $\mathbb{A}$. For simplicity, we treat moment as $S = 1/2$ localized on Os as was done in [6].

At each Na site i, the internal field is given by,

$$H^{i}_{\mathrm{int}} = \hat{\mathbf{h}} \cdot \sum_{\langle j \rangle} \mathbb{A}_{\mathrm{j}} \cdot \boldsymbol{\mu}_{\mathrm{j}} \tag{3}$$

where $\hat{\mathbf{h}}$ is a unit vector in the applied field direction, $\mathbb{A}_{\mathrm{j}}$ the symmetric 3×3 hyperfine coupling tensor with the jth nearest-neighbor Os atom and $\boldsymbol{\mu}_{\mathrm{j}}$ its magnetic moment. We calculate the average local H_{u} and H_{stag} at the Na site as well as Na NMR spectra, which is a histogram of the local field component projected along the applied field, by performing a full lattice sum for different orientations of the applied magnetic field. We fit the data (plotted in Fig. 4 of the main paper) to simulated local fields with magnitude and direction of the local Os moments, and a relative strength of the off-diagonal terms of $\mathbb{A}$ ($A_{\mathrm{ij}}/A_{\mathrm{ii}}$) as fitting parameters. We find that simple canted antiferromagnetic and ferrimagnetic-like orders, that would naturally explain low moments observed in [3, 8], cannot account for our data. Only models that produce spin component perpendicular to the direction of the applied field are consistent with our observations. Among these, canted FM model, recently proposed as an allowed spin model on the *fcc* lattice with tetragonal distortions in [6, 7] and depicted in Fig. 4B, fully accounts for our data as illustrated by the solid lines in Fig. 4A.

This canted FM model consists of two inequivalent sub-lattices with moments in each layer in the XY plane parallel to each other, forming ferromagnetic order, while moments in the neighboring layers point to a different direction. Furthermore, moments in two adjacent layers are symmetric about [110] axis, as shown in Fig. 4B of the paper. That is, the moments are rotated by angle ϕ away from [110] (or applied field direction if moments follow the field) in one layer and by angle $-\phi$ away from [110] in the neighboring layers. Moments arranged in this fashion induce an uniform

magnetic moment in [110] direction, providing an overall shift to the NMR spectrum, and form a staggered pattern in the direction perpendicular to the uniform moment, *i.e.* [110]. By setting this angle as a fitting parameter, we have control over the amplitude ratio between uniform and staggered local fields induced at the Na sites. First, we fix the orientation of the magnetic moments in XY plane and vary angle ϕ, moment amplitude, and hyperfine tensor to fit our data. We find that data (H_{u} and H_{stag} as the applied field is rotated in the $(1\bar{1}0)$ plane), is best accounted for by the following parameters: the angle $\phi \approx 67°$, moment $\mu \approx 0.6\,\mu_B$ (which is the value of the moment deduced from the fit to a Curie-Weiss behavior in the PM state in [3]), and transfer hyperfine coupling tensor in the units of (T/μ_B),

$$\mathbb{A} \approx \begin{bmatrix} 0.45 & -0.13 & -0.14 \\ 0.13 & 0.45 & 0.16 \\ -0.14 & 0.16 & 0.48 \end{bmatrix} (\mathrm{T}/\mu_{\mathrm{B}}). \tag{4}$$

The moment value of $0.6\,\mu_{\mathrm{B}}$ is specified for $\mathbf{H}\|[001]$ and as direction of H is rotated in the $(1\bar{1}0)$ plane, we adjust the value of the moment to scale as bulk magnetization at a given direction of $\mathbf{H}$. In this case, angle ϕ remains independent of the direction of $\mathbf{H}$ and spin remain in-plane that follows the rotation of $\mathbf{H}$. In addition, for our initial guess for diagonal values of $\mathbb{A}$, we used the same values as those determined in the PM state, for reasons described in the previous section. Thus, we constrained the values of diagonal elements of $\mathbb{A}$ to be close to those found in the paramagnetic state. As lattice distortions emerge at low temperature, there is no *a priori* reason to believe that diagonal terms in FM phase should be equal to those found in the PM state. However, we have no independent way of knowing the exact strength of the elements of $\mathbb{A}$, as our data only senses the product of A and μ_{B}. Nevertheless, we can consider two different scenarios. In the first scenario, we assume that the diagonal elements of $\mathbb{A}$ are close to those found in the PM state, as described above, and find that the effective FM moment of $0.6\,\mu_{\mathrm{B}}$, for $\mathbf{H}\|[001]$. This assumption seems reasonable, as the main effect of lattice distortions, which do not exceed 0.8% of the lattice constant, is to induce non-zero off-diagonal terms and not to drastically change values of the diagonal terms of $\mathbb{A}$. The value of so deduced moment corresponds to that found from the bulk magnetization data in the PM state, and is three times larger than that found in the FM state in [3]. Thus, small moment found in the FM state from bulk measurements can be explained by large canting angle ϕ that we determined. That is, small moment is due to partial cancelation of nonparallel magnetic moments.

Alternatively, if moment is assumed to be $0.2\,\mu_{\rm B}$, which is the value of the moment in the FM state as determined from bulk magnetization [3], we find that all the elements of $\mathbb{A}$ displayed in Supplementary Equation 4 are multiplied by a factor of three. Evidently, in either case, we find finite off diagonal terms, induced by the O octahedra charge distribution distortions around Na atoms. We emphasize that symmetry of the inferred $\mathbb{A}$ tensor does not reflect neither local tetragonal nor orthorhombic symmetry of the distorted O octahedra. This implies that spin-spin interactions are mediated by complex multipolar interactions, as theoretically predicted in [6].

As magnetic field is rotated and spin-plane follows $\mathbf{H}$, it is also possible that the moment value remains constant at $0.6\,\mu_{\rm B}$ and it is variation of the angle ϕ that accounts for the observed angular dependence of $H_{\rm u}$ and $H_{\rm stag}$ (and bulk magnetization as well). In this case, we find that the angle ϕ varies from 65° to 71° as $\mathbf{H}$ is rotated from [001] to [110] direction. Moreover, the inferred value of ϕ, allows us to estimate the ratio of in-plane ($J^{'}$) to intra-plane (J) coupling constant [6]. In Ref. [6] the authors deduced that in the canted-FM state spins lie in the (xy) plane with angles given by,

$$\tan\phi_A = -\frac{J^{'}}{4|J|} - \sqrt{1+\frac{J^{'}}{4|J|}}\,, \quad \tan\phi_B = \frac{\pi}{2} - \phi_A, \quad \text{for } J^{'} < 0\,. \tag{5}$$

Even though this result was derived for zero magnetic field, we showed that angle ϕ does not vary significantly on the strength of the applied field, justifying the use of the above expression. Thus, we find that $J^{'}/J \approx 4$.

Furthermore, we see no evidence of domain formation and significant lag of the magnetic moment behind the field. Indeed, in Supplementary Figure 4, we plot integrated spectral intensity, reflecting total number of nuclei, as a function of the angle θ. The facts that variation in the intensity does not exceed 25 % and that the intensity at [001] and [110] is the same, demonstrate that there are no significant domains in which moments are oriented away from the applied field, *i.e.* local magnetization follows the field direction.

Supplementary Note 5: Quadrupolar effects and charge density distortions

Axially Symmetric Case:

In the simplest case of a field with axial symmetry, interaction between eq, the electric field gradient (EFG), and the nucleus, with spin I and the quadrupole moment Q, is described by the

Quadrupole Hamiltonian,

$$\mathcal{H}_{\mathrm{Q}} = \frac{(eQ)(eq)}{4I(2I-1)}[3I_{\mathrm{z}}^2 - I(I+1)]. \tag{6}$$

For nuclear spin $I = 3/2$, as is the case of ^{23}Na, the energy eigenstates of $\mathcal{H}_{\mathrm{Q}}$ are given by,

$$E = \frac{(eQ)(eq)}{4I(2I-1)}\left[3m^2 - I(I+1)\right] \tag{7}$$

Than, the frequencies between different quadrupole satellite transitions is given by,

$$\begin{aligned}\omega_{\mathrm{m}\to\mathrm{m}-1} = \frac{(eQ)(eq)}{h\,4I(2I-1)}\left[3(2m-1)\right] = \ & \frac{1}{2}\frac{(eQ)(eq)}{h}, \quad \text{for} \quad |+3/2\rangle \to |+1/2\rangle \\ & 0\frac{(eQ)(eq)}{h}, \quad \text{for} \quad |+1/2\rangle \to |-1/2\rangle \\ & -\frac{1}{2}\frac{(eQ)(eq)}{h}, \quad \text{for} \quad |-1/2\rangle \to |-3/2\rangle\,. \end{aligned} \tag{8}$$

Furthermore, the splitting δ_{q} between adjacent quadrupole satellites, in a field along the principal axis of the EFG, equals

$$\delta_{\mathrm{q}} = \frac{1}{2h}(eQ)(eq) = \frac{1}{2h}(\text{Quadrupole moment}) \times (\text{EFG}). \tag{9}$$

Evidently, in this case equal splitting is observed between quadrupole satellites lines, as observed in our experiment. Further, we can estimate the value of the EFG using experimentally determined value of the splitting, that is $\delta_q \approx 190\,\mathrm{KHz}$ for $H\|[001]$,

$$(EFG) = \frac{2h\delta_q}{eQ} = \frac{2 \times 4.136 \times 10^{-15}\,\mathrm{eV} \cdot s \times 190 \times 10^3\,\mathrm{s}^{-1}}{0.12 \times e \times 10^{-28}\,\mathrm{m}^2} = 1.31 \times 10^{20}\,\mathrm{V/m}^2. \tag{10}$$

Next, this value can be used to estimate particular lattice distortions in our material. In oxygen octahedra surrounding Na nuclei the EFG takes on the following form,

$$EFG = \frac{2q}{4\pi\epsilon_0}\begin{bmatrix} \frac{2}{a^3} - \frac{1}{b^3} - \frac{1}{c^3} & 0 & 0 \\ 0 & -\frac{1}{a^3} + \frac{2}{b^3} - \frac{1}{c^3} & 0 \\ 0 & 0 & -\frac{1}{a^3} - \frac{1}{b^3} + \frac{2}{c^3} \end{bmatrix} \tag{11}$$

Clearly, in materials with cubic symmetry, such as Ba_2NaOsO_6 in the paramagnetic state, $a = b = c$ so that the EFG equals zero, which leads to vanishing splitting, δ_{q}.

The observed δ_{q} is the largest for field applied in the [001] direction, as shown in Fig. 2 of the manuscript. In this case the simplest model, accounting for the splitting of the Na line into three equally spaced quadrupole satellite lines, involves distortions of the O octahedra surrounding Na

nuclei solely along the [001] direction. In this case, $q = 2e$, $a = b \neq c$, and we obtain

$$EFG = \frac{2q}{4\pi\epsilon_0}\begin{bmatrix} \frac{1}{a^3} - \frac{1}{c^3} & 0 & 0 \\ 0 & \frac{1}{a^3} - \frac{1}{c^3} & 0 \\ 0 & 0 & -2(\frac{1}{a^3} - \frac{1}{c^3}) \end{bmatrix}. \tag{12}$$

Therefore, the principal axis of the EFG ($V_{zz} \equiv eq$) is given by,

$$V_{\mathrm{zz}} = \pm\frac{8e}{4\pi\epsilon_0}\left(\frac{1}{a^3} - \frac{1}{c^3}\right) \tag{13}$$

$$\left(\frac{1}{a^3} - \frac{1}{c^3}\right) = \pm\frac{4\pi\epsilon_0}{8e} \times 1.31 \times 10^{20}\,\mathrm{V/m^2} = \pm\,0.01137 \times 10^{30}\,\mathrm{m^{-3}}. \tag{14}$$

In Ba_2NaOsO_6 with $a = 2.274$ Å, distortions along the c crystalline axis of the order of 4 % can account for the observed δ_q, that is

$$\begin{aligned} &\frac{1}{c^3} = \frac{1}{a^3} \pm 0.01137 \\ &c = 2.181\text{Å} \quad (-4.1\%), \quad \text{for compression} \\ &c = 2.385\text{Å} \quad (4.9\%), \quad \text{for elongation.} \end{aligned} \tag{15}$$

Including Anisotropy:

Quadrupole Hamiltonian expressed in the coordinate system define by the principal axes of the EFG, is given by

$$\mathcal{H}_{\mathrm{Q}}(x,y) = \frac{eQV_{zz}}{4I(2I-1)}\left[(3\hat{I}_z^2 - \hat{I}^2) + \eta(\hat{I}_x^2 - \hat{I}_y^2)\right], \tag{16}$$

where $\eta \equiv |V_{xx} - V_{yy}|/V_{zz}$ is asymmetry parameter and V_{xx}, V_{yy}, and V_{zz} are diagonal components of EFG. Here, V_{zz} is defined as the principle component of the EFG and $|V_{\mathrm{xx}}| < |V_{\mathrm{yy}}| < |V_{\mathrm{zz}}|$, by convention. In this case, the splitting is given by,

$$\delta_{\mathrm{q}} = \frac{(eQ)(V_{\mathrm{zz}})}{2h}\left(1 + \frac{\eta^2}{3}\right)^{1/2}. \tag{17}$$

Thus, the value of δ_{q} is dictated by both V_{zz} and anisotropy parameter. To find the full set of possible distortions that can account for our observations, that $\delta_{\mathrm{q}} \approx 190\,\mathrm{KHz}$ for all satellite transitions, we used the point charge model to calculate V_{zz} and η. In this model the electron density at the Na site is calculated by taking all the surrounding charges, which are treated as the point charges of zero radius that carry the appropriate ionic charge, into an account.

In addition to the absolute value of the splitting δ_{q}, we also measure the angular dependence of the splitting as the applied magnetic field is rotated by angle (θ) with respect to the crystalline axis. In the high field limit, when Quadrupolar Hamiltonian is a perturbation to the dominant Zeeman term, the angular dependence of the splitting is given by

$$\delta_{\mathrm{q}} = \frac{\Delta_{\mathrm{q}}}{2}(3\cos^2\theta - 1 + \eta\sin^2\theta\cos 2\phi), \tag{18}$$

where θ is the angle between the applied field $\mathbf{H}$ and V_{zz} (see Supplementary Figure 5), and $\eta = |V_{\mathrm{xx}} - V_{\mathrm{yy}}|/V_{\mathrm{zz}}$ the asymmetric factor. Our measurements of the angular dependence of δ_{q}, plotted in Fig. 2B, as $\mathbf{H}$ is rotated from [001] to [110], can be accounted for by two models for the reasons we describe next.

As shown in Fig. 2B, we observe no more than 3 lines per set (I or II) regardless of the angle θ. This indicates that the principal axes of the EFG coincide with those of the crystal. In a material with cubic symmetry, it is thus possible to stabilize three different domains, each with the principle axis of the EFG, V_{zz}, pointing along any of the 3 equivalent crystal axes. Further, local magnetic field has to be parallel to V_{zz} in each domain. The facts that the splitting is the largest for $H\|[001]$ (Fig. 2B), and that only 3 peaks per set are observed for $\mathbf{H}\|[110]$ imply that two domains are plausible in the crystal. One domain is characterized by pure uniaxial $3z^2 - r^2$ distortions where V_{zz} is in [001] direction, while the other is distinguished by $x^2 - y^2$ distortions where V_{zz} is then in the (110) plane. In the simplest model V_{zz} is aligned along [001] direction, as illustrated in Supplementary Figure 5(a), $\eta = 0$, and $\phi = 45°$, in which case, $\delta_{\mathrm{q}} = \frac{\Delta_{\mathrm{q}}}{2}(3\cos^2\theta - 1)$. In the second model V_{zz} is aligned along [100] direction, as illustrated in Supplementary Figure 5(b) and η is of the order of 1. Thus, distortions that generate both tetragonal and orthorhombic local symmetry at the Na site can account for the data. In fact, we find that distortions for which V_{zz} is in [001] direction are tetragonal, while for orthorhombic distortions V_{zz} may be in the (110) plane, in which case $x^2 - y^2$ type distortions are dominant rather than pure uniaxial $3z^2 - r^2$ ones. Combining the above facts with the point charge calculations, we find one class of possible distortion that satisfy all the constraints imposed by our data. This class comprises of orthorhombic distortions for which $\eta \approx 0.95$.

SUPPLEMENTARY DISCUSSIONS

Supplementary Discussion 1: Microscopic model of lattice and/or charge density distortions

We found that our observation, revealing equal δ_q on two magnetically inequivalent Na sites, can be explained by one possible scenarios involving distortions of the O^{2-} octahedra surrounding Na^+ ions as depicted in Supplementary Figure 6. In this model, we allow the oxygen to move by different amounts along each of the three axes and so constrain it to the cubic axes of the perovskite reference unit cell, as suggested in [7]. Only, one structurally distinct Na site is induced by the distortion. Moreover, we point out that we cannot distinguish between displacement of the actual O atoms and distortions of the O charge density.

In this model, only one structurally distinct Na sites is generated, as depicted in Supplementary Figure 6. As described above, such distortions of the oxygen tetrahedra comprise of either dominant tetragonal distortions along [001] direction, for which $\eta = 0$, or orthorhombic distortions for which $\eta \approx 0.95$. However, we emphasize that only the orthorhombic distortions for which $\eta \approx 0.95$ can account for both the amplitude of the observed splitting and its dependence on the field orientation in the low temperature FM phase. Our calculations indicate that no distortion along any particular direction that exceeds 0.8% of the respective lattice constant, can induce detected splitting of ≈ 190 kHz. Furthermore, we find several possible distortions which can induce the observed splitting. That is, different combinations of compression and/or elongation, and their respective amplitudes, along any particular axis can induce the observed splitting. However, the evolution of the splitting as a function of the direction of applied field in the different planes of the crystal, restricts possible distortions to those with an orthorhombic point symmetry. At higher temperatures, outside the LRO phase, the analysis of the width of the NMR spectra allows us to place an upper limit on the magnitude of the distortions. Because we do not observe the well defined splitting, its exact dependence on the field orientation is unknown. Therefore, in principle dominant tetragonal distortions can account for the line broadening. Lastly, we point out that this model, leads to only one magnetically distinct Na sites. Therefore, the observed magnetically distinct Na sites, *i.e.* sites sensing two different local fields, do not arise from lattice distortion as an artifact of NMR performed on the nuclei that does not carry electronic spin.

Supplementary Discussion 2: Mechanisms of lattice distortions in perovskite oxides

It is well known that perovskite oxides are prone to lattice distortions [9, 10]. In this section we describe why common lattice instabilities often present in perovskite transition metal oxides are not compatible with our observations. These lattice distortions involve changes in symmetry and global detectable change of lattice parameters. Moreover, a typical distortion mechanism involves a tilting of essentially rigid oxygen polyhedra, as is the case in $GdFeO_3$. These distortions would induce EFG with the principal axes that do not align with those of the crystal. In this case, the NMR spectra will not show three peaks (per magnetic site, I and/or II) for every orientation of the applied magnetic field, as was the case in our experiment. Evolution of both NMR spectral shapes and the magnitude of the splitting (δ_q) as the direction of the applied field is rotated with respect to crystal axes is compatible with the EFG, whose principal axes align with those of the crystal. Such EFG at the Na site can only be induced by allowing the oxygen to move by different amounts along each of the three crystal axes, *i.e.* the cubic axes of the perovskite reference unit cell, as was suggested to be the case for lattice distortions induced by the quadrupolar mechanism in [7]. We have performed detailed point charge calculations of the EFG and δ_q considering possible tilting of the oxygen octahedra. We found that tilting of the rigid (undistorted) oxygen octahedra is not compatible with our NMR observations. Furthermore, typical values of the bent in the bond involving oxygen in $GdFeO_3$ are roughly between 145-170°. Our NMR data cannot be accounted for by such large bond bending.

However, if we consider the possible tilt angle of the distorted oxygen octahedra, as depicted in Fig.2c of the manuscript, our data places un upper bound on the tilt angle. We find that the tilt angle cannot exceed 4°. Such tilting will induce displacement of oxygen ions that is much smaller than the dominant displacement along the cubic axes of the perovskite reference unit cell. We emphasize, that our observations can only be accounted for by considering the dominant displacement along the cubic axes of the perovskite reference unit cell with possible tilt of the oxygen octahedra that cannot exceed 4°.

Furthermore, the structural change that we infer and report induces only one structurally inequivalent Na site, while the $GdFeO_3$ type distortions of the double perovskite structure produce two inequivalent Na sites. Such structural distortions leading to one inequivalent Na site, do not lead to two magnetically inequivalent Na sites in a trivial way. Two magnetic sub-lattices with FM moments pointing in different directions are required to account for our data.

Supplementary Discussion 3: Entropy considerations

Including SOC effects, the anticipated ground state of Ba_2NaOsO_6 for a perfectly cubic point symmetry is predicted to be $J_{\text{eff}} = \frac{3}{2}$. However, the magnetic entropy removed across FM transition is only $R\ln 2$ [3]. The quantum model approach predicts that the additional entropy of $R\ln 2$ must be removed by quadrupole or orbital ordering. Thus, one can naively expect that the large entropy change of $R\ln 4$ should be observed around T_c, since the onset temperature for the broken local point symmetry (BLPS) phase (that is, quadrupolar ordering) is very close to the magnetic one, in disagreement with the entropy removal of $R\ln 2$ reported in Ref. [3]. Resolution of this apparent disagreement is unveiled by determination of the true onset temperature for quadrupolar order, which is much higher than the onset temperature for breaking of local cubic symmetry reported by NMR.

Onset temperature for breaking of local cubic symmetry, shown in Fig. 1b of the main manuscript, was identified as temperature below which the second moment of the NMR spectral line, measuring the spectral width, increases notably as compared to that in high temperature PM phase. We point out that this temperature does not necessarily correspond to the true onset temperature for orbital ordering, but to temperature below which lattice distortions do not exceed resolution of our experiment, that equals to 0.02% of the respective lattice constant. As a matter of fact, guided by our NMR findings of breaking of local cubic symmetry, high resolution x-ray scattering experiments have detected tetragonal distortions at much higher temperatures [11]. That is transition from a cubic to tetragonal local symmetry has been observed at 320 K [11]. The tetragonal distortions do not exceed 0.01% of the respective lattice constant at temperatures in the vicinity of 100 K, which is beyond resolution of 0.02% in our ^{23}Na NMR measurements. Therefore, the true onset temperature for the quadrupolar ordering, *i.e.* breaking of the local cubic symmetry, is much higher than T_{c} associated with magnetic LRO, as predicted in Ref. [6, 7, 12]. Consequently, the missing entropy is distributed over a wide temperature range of the order of 300 K.

We deduced that in the LRO phase, breaking of the local cubic symmetry is caused by orthorhombic distortions. In the PM phase, we found that it is possible that pure uniaxial tetragonal lattice distortions, along [001] direction, could be present above the magnetic transition. This is in agreement with observed tetragonal distortions at higher T by x-ray scattering [11]. The associated tetragonal-to-orthorhombic phase transition could then be related to a nematic phase transition that was recently proposed in Ref. [6], based on the same quantum model approach. As a matter of fact, nematic phase transition that was recently proposed in Ref. [6] refers to or-

thorhombic (in the PM phase)-to-tetragonal phase transition. However, the same physics may lead to tetragonal (in the PM phase)-to-orthorhombic phase transition. This is because the authors in Ref. [6] assumed that the low temperature distorted phase has tetragonal symmetry and studied magnetism as a spin $S = 1/2$ model with general exotic directional magnetic interaction on an fcc lattice. In Ba_2NaOsO_6 an effective $S = 3/2$ spin resides on an fcc lattice. This large spin may still be quantum, that is, may be reduced to an effective $S = 1/2$, by unusual multipolar interactions, that are basis of quantum models of magnetism in Mott insulators with strong SOC, and/or a structural transition or quadrupolar ordering [7]. By assuming that tetragonal distortions are responsible for reducing the effective spin to $S = 1/2$, the authors in Ref. [6] investigated magnetism in double perovskites with strong SOC (implying complex directional interactions), such as Ba_2NaOsO_6. The key point is that this model correctly predicts essential features of LRO magnetism in Ba_2NaOsO_6, which is that there is a site-differentiated magnetic structure. In addition, the authors found that spin-lattice coupling can give rise to an intermediate temperature paramagnetic nematic/orthorombic phase. The details of the the transition to/from this phase is predominantly controlled by the strength of the spin-lattice coupling. If one assumes that orthorhombic distortions are present in the magnetic state it is possible that spin-lattice coupling can give rise to an intermediate temperature paramagnetic nematic/tetragonal phase.

SUPPLEMENTARY REFERENCES

[1] Abragam, A. *"Principles of Nuclear Magnetism"*, 216-263 (Oxford University Press, New York, 1996).

[2] K. E. Stitzer, M. D. Smith, H.-C. zur Loye. *Crystal growth of Ba_2MOsO_6 (M = Li, Na) from reactive hydroxide fluxes. Solid State Sci.*. **4**, 311-316 (2002).

[3] A. S. Erickson, S. Misra, G. J. Miller, R. R. Gupta, Z. Schlesinger, W. A. Harrison, J. M. Kim, and I. R. Fisher. *Ferromagnetism in the Mott insulator Ba_2NaOsO_6. Phys. Rev. Lett.*, **99**, 016404 (2007).

[4] A.M. Clogston, V. Jaccarino, and Y.Yafet. *Interpretation of Knight Shifts and Susceptibilities of Transition Metals: Platinum.* Phys. Rev. **135**, A650-A661 (1964).

[5] S. Gangopadhyay and W. E. Pickett. *Spin-orbit coupling, strong correlation, and insulator-metal transitions: The $J_{eff} = 3/2$ ferromagnetic Dirac-Mott insulator Ba_2MOsO_6*. Phys. Rev. B, **91**, 045133 (2015).

[6] Hiroaki Ishizuka and Leon Balents. *Magnetism in $S = 1/2$ double perovskites with strong spin-orbit interactions.* Phys. Rev. B, **90**, 184422 (2014).

[7] G. Chen, R. Pereira, and L. Balents *Exotic phases induced by strong spin-orbit coupling in ordered double perovskites.* Phys. Rev. B, **82**, 174440 (2010).

[8] A. J. Steele, P. J. Baker, T. Lancaster, F. L. Pratt, I. Franke, S. Ghannadzadeh, P. A. Goddard, W. Hayes, D. Prabhakaran, S. J. Blundell, *Low-moment magnetism in the double perovskites Ba_2MOsO_6 (M=Li, Na).* Phys. Rev. B, **84**, 144416 (2011).

[9] K. S. Aleksandrov and J. Bartolome, *Structural distortions in families of perovskite-like crystals.* Phase Transition **74**, 255-336 (2001).

[10] J.-S. Zhou and J. B. Goodenough, *Intrinsic structural distortion in orthorhombic perovskite oxides.* Phys. Rev. B **77**, 132104 (2008).

[11] Z. Islam and I. R. Fisher. (private communications).

[12] William Witczak-Krempa, Gang Chen, Yong Baek Kim, and Leon Balents. *Correlated quantum phenomena in the strong spin-orbit regime.* Annual Review of Condensed Matter Physics, **5(1)**, 57-82 (2014).
